# Preparation of Monodisperse Enrofloxacin Molecularly Imprinted Polymer Microspheres and Their Recognition Characteristics

**DOI:** 10.1155/2019/5970754

**Published:** 2019-04-01

**Authors:** Xiaoxiao Wang, Yanqiang Zhou, Yuling Niu, Shanwen Zhao, Bolin Gong

**Affiliations:** ^1^College of Chemistry and Chemical Engineering, North Minzu University, Yinchuan 750021, China; ^2^Ningxia Entry-Exit Inspection and Quarantine Bureau Comprehensive Technology Center, Yinchuan, Ningxia, China

## Abstract

This study presents a new strategy for the detection of enrofloxacin (ENR) in food samples by the use of monodisperse ENR molecularly imprinted polymers (MIPs). Using enrofloxacin as template molecule, methacrylic acid as functional monomer, and ethylene diglycidyl ether as cross-linker, surface molecularly imprinted polymers (MIPs) were prepared on the surface of polymeric glycidyl methacrylate-ethylene glycol dimethacrylate (P_GMA-EDMA_) microspheres. The surface morphology and imprinting behavior of P_GMA-EDMA_@MIPs were investigated and optimized. Synthesized P_GMA-EDMA_@MIPs showed good physical and chemical stability and specific recognition toward fluoroquinolones. The introduction of P_GMA-EDMA_ microspheres greatly increased the adsorption area of P_GMA-EDMA_@MIPs and increased the adsorption capacity of target molecules. The core shell structure increased the adsorption rate, and adsorption equilibrium was achieved within 6 min, much higher than that of MIPs synthesized by traditional methods. Enrofloxacin in milk samples was detected by molecular imprinting solid phase extraction (MISPE) combined with high performance liquid chromatography (HPLC). Implementing this method resulted in a recovery rate of 94.6~109.6% with a relative standard deviation (RSD) of less than 3.2%. The limit of detection (LOD) of this method was identified as three times the signal-to-noise ratio (10 *μ*g/L). In summary, this work proposed a sensitive, rapid, and convenient method for the determination of trace ENR in food samples.

## 1. Introduction

Enrofloxacin (ENR) is an antibiotic belonging to the fluoroquinolone class (FQs) of synthetic antibiotics. The physicochemical properties and broad spectrum of activity of FQs antibiotics against most bacteria have led to their widespread use in human medicine, as well as the prevention and treatment of animal diseases in livestock [1–3]. However, trace amounts of these drugs can be detected in food products taken from animals treated with FQs antibiotics, posing a threat to human health (such as toxicity, drug resistance, and anaphylaxis) [4]. In fact, many countries have established a maximum limit of FQs residues in food. Additionally, the environmental impact of FQs residues has recently attracted worldwide attention. Therefore, sensitive and selective method for the detection and quantification of FQs is an important area of research. The currently available methods for the detection of FQs in different environments consist of high performance liquid chromatography [5] coupled to mass spectrometry [6, 7], ultraviolet (UV) [8], and fluorescence detection[9, 10]. However, the complex matrices of biological samples present challenges for the selective quantification of trace amounts of FQs in food products.

Molecularly imprinted technology (MIT) [11–13] is a method that can be used to develop polymers for the selective identification of a particular molecular target or a class of molecules [14]. To prepare the polymer, a functional monomer is constructed with binding sites matching the shape, size, and functional groups of a template molecule. The functional monomers are then copolymerized and cross-linked in the presence of the template molecule to create a polymer network capable of molecular recognition. Subsequent removal of the template molecules from the polymer network facilitates the selective binding and recognition of the template molecule or structural analogs in a sample mixture [15]. MIPs are attractive materials for molecular recognition due to their low cost, simple preparation, high stability, and good reproducibility under harsh chemical and physical conditions. Consequently, MIT has been widely used in extraction separation [16, 17], chemical sensing [18], catalysis [19], and chiral separations [20–22]. Surface molecular imprinting distributes almost all binding sites on a surface with good accessibility by taking some measures, which facilitates the removal and recombination of template molecules. This method is therefore particularly suitable for the preparation of imprinted polymers of biomacromolecules. The distribution of binding sites across the polymer surface shortens the adsorption equilibrium time by overcoming the traditional embedding phenomenon [23, 24].

The preparation of MIPs using ENR as a template molecule has been recently explored in the literature; the resulting MIPs would have important implications for the selective detection of ENR in complex matrices. Xiao* et al.* [25] prepared FQs imprinted polymers on the surface of magnetic carbon nanotubes and simultaneously used a pseudo template to achieve fluoroquinolone extraction. Additionally, work by He* et al.* [26] firstly attempted to prepare an ENR imprinted material using the magnetic polyhedral oligomeric semi-siloxane composite as a matrix. In a similar vein, Yan* et al.* [27] thermally initiated polymerization of the monolithic column and use norfloxacin as a pseudo template for ENR; the resulting material demonstrated a high affinity for ENR and norfloxacin in water and successfully afforded the extraction of ENR and norfloxacin from blood samples. Despite these great advancements in the development of MIPs, the preparation of monodisperse ENR imprinted polymers by surface grafting and the application of the resultant polymers to a real sample analysis have yet to be reported.

In this work, self-made monodisperse macroporous cross-linked polymeric glycidyl methacrylate-ethylene glycol dimethacrylate (P_GMA-EDMA_) microspheres are used as the matrix. Compared with silica gel and other carriers, it is more resistant to acid and alkaliand has a large number of active groups on the surface. It is an ideal molecularly imprinted carrier. A surface grafting technique was used to prepare enrofloxacin molecular imprinted polymers attached to the surface of P_GMA-EDMA_ microspheres via MPS modified sites. The surface structure and the physicochemical properties of the polymers were analyzed and used for the detection of four FQs in milk samples.

## 2. Materials and Method

### 2.1. Reagent and Instrument

Enrofloxacin (ENR), tetracycline (TC), ofloxacin (OFL), chloramphenicol (CAP), glycidyl methacrylate (GMA), ethylene glycol dimethacrylate (EDMA), ethylene glycol diglycidyl ether (EGDE), methacrylic acid (MAA), ammonium persulfate [(NH_4_)_2_S_2_O_8_], 3-methacryloylpropyl trimethoxysilane (MPS), styrene (St), azobisisobutyronitrile (AIBN), polyvinylpyrrolidone (PVP), cyclohexanol, poly(vinyl alcohol) (PVA), dibutyl phthalate (DBP), ethyl alcohol, pyridine, methanol, acetone, acetic acid, sodium dodecyl sulfate(SDS),and tetrahydrofuran (THF) were purchased from Aladdin Reagent (Shanghai, China). HPLC grade methanol and acetonitrile were obtained from Sigma (St. Louis, MO, USA). Milk samples were purchased from a local supermarket. The polymerization inhibitor was removed from the MAA by using a vacuum distillation unit. Water was twice distilled prior to use. All other reagents were of analytical grade and used without further purification unless otherwise specified. All solutions prepared for HPLC were filtered through a 0.45*μ*m nylon filter before use.

Chromatography was performed using an LC-20AT chromatographic system (Shimadzu, Japan) equipped with two LC-20AT pumps and a SPD-20A UV–VIS detector. Samples were injected through a Rheodyne 7725 valve. Polymers morphology was characterized by using a JSM-7500F electron scanning microscope (JEOL Co., Japan). Elemental analysis was performed on a VarioEL III elemental analyzer (Elementar Co., Germany). Infrared spectra were collected by using a Fourier transform infrared spectrometer (Shimadzu, Japan). Absorption spectra were collected by using a TU-1810-type ultraviolet spectrophotometer (Beijing General Instrument Co., Ltd., China). Centrifugation was performed with a TG16-WS high-speed centrifuge (Centrifuge Factory, China).

### 2.2. Preparation of Monodisperse *P*_*GMA*-*EDMA*_ Microspheres 

Monodisperse polystyrene seeds (PS) were synthesized according to a dispersion polymerization method. The monomer styrene (10 mL), initiator ABIN (0.2 g), stabilizer PVP (2 g), and anhydrous ethanol (87.8 mL) were combined in a 100 mL single-necked flask and sonicated until all of the styrene, ABIN, and PVP were dissolved. The flask was then attached to a rotary device equipped with a heating apparatus. Polymerization was carried out at 70°C over the course of 24 h. Upon completion of the reaction, the solvent was removed by centrifugation and the remaining solids were washed with copious amounts of ethanol to obtain the desired PS.

In a 150 mL single-necked flask, GMA (6 mL), ABIN (0.36 g), EDMA (6 mL), DBP (6 mL), and cyclohexanol (6 mL) were combined and sonicated to dissolve completely; then 45 mL distilled water, 75 mL 0.2% SDS solution, and 35 mL 5% PVA solution were added to the above mixed solution. The reaction mixture was subjected to ultrasonic emulsification for 30 min until complete emulsification of the organic phase was achieved. The resultant emulsion was slowly added to a solution of the prepared monodisperse PS while the temperature was maintained at 30°C. The reaction mixtures were stirred for 24 h, then degassed under a nitrogen atmosphere for 20 min, and still stirred at 70°C for additional 24 h. The solids obtained were washed with water, methanol, and acetone and then dried in vacuo to yield the product. The porogens were removed by extraction with tetrahydrofuran for 48 h in a Soxhlet apparatus for products. The products were washed with methanol again and dried in vacuo to yield the P_GMA-EDMA_ microspheres.

P_GMA-EDM_ (2.0 g) were suspended in 100 mL of 0.1 mol/L sulfuric acid, stirred, and kept at 60°C for 12 h. Then the products were filtered and washed with water until neutral and then dried under vacuum to afford the hydrolyzed P_GMA-EDMA_ microspheres.

### 2.3. Preparation of *P*_*GMA*-*EDMA*_@MIPs

#### 2.3.1. Bonding of MPS on the Surface of *P*_*GMA*-*EDMA*_

The hydrolyzed microspheres P_GMA-EDMA_ (2.0 g) were ultrasonically dispersed in 100 mL of 50% (V/V) ethanol. MPS (2.5 mL) and pyridine (3 drops) were added to the dispersion and the mixtures were heated to 50°C for 24 h. Unreacted silylation reagent was removed from the mixture by vacuum filtration using absolute ethanol. The products were concentrated in vacuo to obtain the modified microspheres P_GMA-EDMA_@MPS.

#### 2.3.2. Grafting MAA on *P*_*GMA*-*EDMA*_@MPS

The P_GMA-EDMA_@MPS (2.0 g) microspheres and MAA (7 mL) were combined in 150 mL of water and (NH4)_2_S_2_O_8_ (0.042 g) were added as an initiator for the graft polymerization reaction. The reactant mixtures were heated to 70°C for 24 h, while constantly stirring under an atmosphere of nitrogen. The resultant microspheres were purified by Soxhlet extraction in ethanol to remove unreacted MAA physically attached to the microspheres. The solution was dried in vacuo to yield the grafted P_GMA-EDMA_@MPS@MAA microspheres.

#### 2.3.3. Preparation of MIPs

The P_GMA-EDMA_@MPS@MAA (2.0 g) microspheres were dissolved in 50mL 10 mmol/L ENR methanol solution. The mixtures were shaken at 25°C for 6 h until the adsorption of ENR by P_GMA-EDMA_@MPS@MAA reached equilibrium. The reactant mixtures were then filtered and the ENR-adsorbed microspheres P_GMA-EDMA_@MPS@MAA were dried under vacuum. The ENR-adsorbed P_GMA-EDMA_@MPS@MAA microspheres (2.0 g) were added to 50mL 4 mmol/L ENR 50 % aqueous methanol solution. The pH of the solution was adjusted to 8.0 using NaOH solution and then EGDE (2.5 mL) was added as a cross-linker. The reactant mixture was stirred at 50°C for 8 h to complete polymerization. After the reaction finished, the product was washed with methanol and dried at 60°C. The MIP material was extracted with Soxhlet using methanol/acetic acid (9/1, v/v) mixture to remove the unreacted cross-linker EGDE and residual template. The non-imprinted polymers (P_GMA-EDMA_@NIPs) were synthesized according to the same procedures described above except in the absence of a template molecule.

### 2.4. Adsorption Experiments

To measure the adsorption capacity of the polymers, the P_GMA-EDMA_@MIPs (20 mg) was mixed with a series of methanol solutions of ENR at various concentrations. Each reactant mixture was shaken for 12 h and then subjected to centrifugation. Supernatant was quantified by UV spectrometry at 280 nm and then the adsorption amount was calculated according to

(1) $$\begin{array}{c} Q=\frac{\left(C_{0}-C_{e}\right)V}{m} \end{array}$$

where* Q* (mg/g) represents the mass of ENR adsorbed per gram of polymer, *C*_o_ (mg/L) and *C*_e_ (mg/L) are the initial and final concentrations of ENR in solution, respectively,* V* (L) is the total volume of the solution, and* m* (g) is the mass of polymer.

For the kinetic experiments, the P_GMA-EDMA_@MIPs (20 mg) was added to a methanol solution of ENR (10 mL, 2 mM). The series of prepared reaction mixtures were mechanically shaken for different lengths of adsorption time at room temperature, after which each of the mixtures was subjected to centrifugation to afford separation. Supernatant was quantified by UV spectrometry at 280 nm and then the adsorption amount was calculated according to (1).

The operation procedures of P_GMA-EDMA_@NIPs were the same as those of P_GMA-EDMA_@MIPs.

### 2.5. Selective Adsorption Experiments

**Figure 1:**
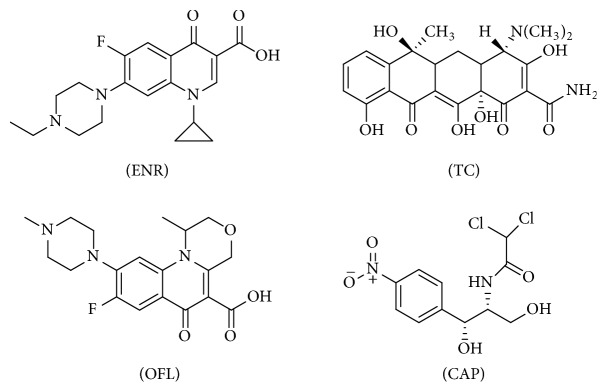
The structures of ENR, TC, OFL, and CAP.

To investigate the selectivity of the prepared P_GMA-EDMA_@MIPs towards ENR, the binding of ENR was tested in comparison to three structural analogs: ofloxacin (OFL), tetracycline (TC), and chloramphenicol (CAP) (Figure 1). The P_GMA-EDMA_@MIPs (20 mg) was added to flasks containing methanol solutions (10 mL, 2 mM) of ENR. The shaking adsorption process was carried out for 12 h at 25°C. The adsorption capacity of P_GMA-EDMA_@MIPs to OFL, TC, and CAP was determined in the same way. The operation procedures of P_GMA-EDMA_@NIPs were the same as those of P_GMA-EDMA_@MIPs.

The distribution coefficients (*K*_*D*_), selectivity coefficients (*k*), relative selectivity coefficients (*k*′), and imprinting factor (*α*) of OTC and CTC with respect to TC can be obtained according to the following equations:

(2) KD=QeCe

(3) k=KDFQsKDTC

(4) k′=KMIPsKNIPs

(5) α=QMIPsQNIPs

where* Q*_*e*_ (mg/g) and* C*_*e*_ (mg/L) represent the amount of binding and concentration of substrate at equilibrium, respectively.* K*_*D(FQS)*_ represents the distribution coefficients of the fluoroquinolones,* K*_*D(TC)*_ represents the distribution coefficients of the tetracyclines, and* K*_*MIPs*_ and* K*_*NIPs*_ are the selectivity coefficients of the P_GMA-EDMA_@MIPs and P_GMA-EDMA_@NIPs, respectively.* Q*_*MIPs*_ and* Q*_*NIPs*_ represent the adsorption capacity of the P_GMA-EDMA_@MIPs and P_GMA-EDMA_@NIPs for ENR, respectively.

### 2.6. Spiked Recovery Experiments and Analysis of Actual Samples

A sample of milk (0.5 mL) solution was combined with acetonitrile (0.5 mL) solution homogenized. This mixture solution contained ENR and its concentration was 0.025 mmol/L. The samples were centrifuged for 5 min at a speed of 10000 rpm. The supernatant was collected and diluted with phosphate buffer solution (PBS, PH 6, 0.25 mM) to 10 mL.

A SPE column filled with P_GMA-EDMA_@MIPs or P_GMA-EDMA_@NIPs was activated with methanol (2 mL) and pure water (2 mL), successively. The spiked milk sample (3 mL) was flowed through the column, and then a methanol/acetic acid (9/1, v/v) solution (3 mL) was used to elute the extracted analytes. The collected eluate was concentrated by N_2_ stream and then dissolved again with 1 mL mobile phase. Finally, 20 *μ*L samples were detected by HPLC (LC-20AT, Shimadzu corporation). Chromatographic conditions: stationary phase: C_18_ reversed phase chromatographic column (150 mm×4.6 mm, Agilent Corporation, USA); Mobile phase: 0.025mol/L phosphoric acid solution (adjust PH to 3.0 with triethylamine.) (A)~acetonitrile (B). Flow rate: 0.8 mL/min; The detection wavelength is 278 nm by UV detector.

## 3. Results and Discussion

### 3.1. Preparation of Enrofloxacin-Imprinted *P*_*GMA*-*EDMA*_@MIPs

**Figure 2:**
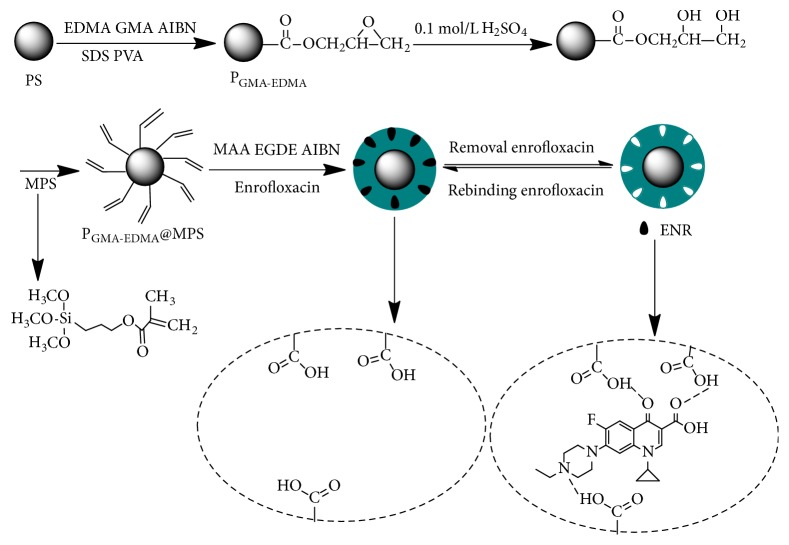
Schematic expression of the reaction process used to prepare P_GMA-EDMA_@MIPs.

To introduce polymerizable double bonds to the surface of the P_GMA-EDMA_ microspheres for facile preparation of the P_GMA-EDMA_@MIPs, the microsphere surface was modified with coupling agent MPS. Polymerization was initiated to graft the functional monomer MAA to the surface of microspheres, and the resultant P_GMA-EDMA_@MPS@MAA microspheres were saturated to adsorb ENR. By adding the cross-linker EGDE, to the microspheres under alkaline conditions, a ring-opening reaction between the epoxy group on the cross-linking agent and the carboxyl group on the P_GMA-EDMA_@MPS@MAA macromolecule forms a network, which encapsulated the ENR molecule, and thereby gained ENR molecularly imprinted polymer. The ENR was then extracted from the P_GMA-EDMA_@MIPs using a methanol/acetic acid (9/1, v/v) mixture, creating holes in the thin layer of the polymer well matched for the size and intermolecular interactions required for ENR binding. The preparation of the P_GMA-EDMA_@MIPs is summarized in Figure 2.

### 3.2. Characterization of Enrofloxacin-Imprinted *P*_*GMA*-*EDMA*_@MIPs

**Figure 3:**
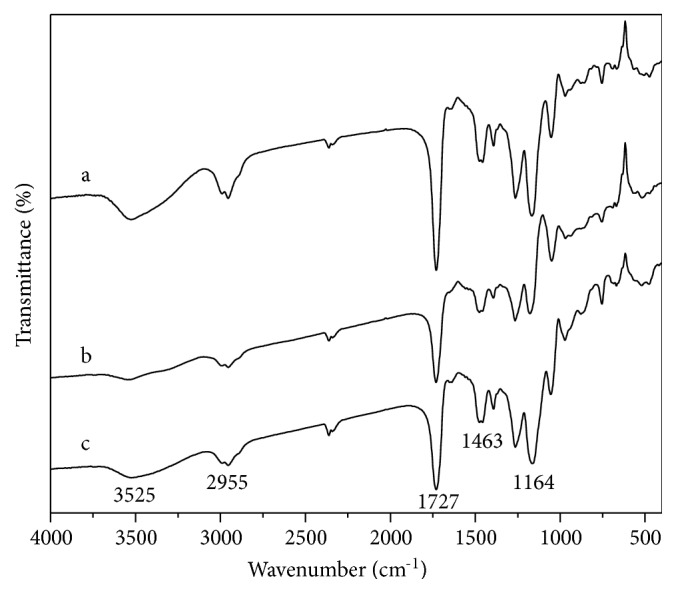
FT-IR spectra of P_GMA/EDMA_ (a), P_GMA/EDMA_@MPS (b), and P_GMA-EDMA_@MIPs (c).

FT-IR analysis of P_GMA-EDMA_@MIPs and the precursor materials is shown in Figure 3. The hydrolyzed P_GMA-EDMA_ microspheres exhibit a strong absorption peak at 1727 cm^−1^ corresponding to the stretching vibration of carbonyl grafted to the microsphere, characteristic -CH stretching vibrations are noted at 2955 cm^−1^, and an -OH stretching vibration is noted at 2955 cm^−1^ as a broad absorption peak. The preparation of P_GMA-EDMA_@MPS depends on the reaction of the silanylated reagent, MPS with hydroxyl groups of the microsphere surface; the observed decrease in the intensity of the characteristic -OH peak at 3525 cm^−1^ in P_GMA-EDMA_@MPS relative to the precursor microspheres is attributed to the partial consumption of the surface -OH functional groups. The spectra of the P_GMA-EDMA_@MIPs show an increase in intensity of a peak around 1093 cm^−1^, corresponding to -C-O-C stretching vibrations in the EGDE cross-linker. Moreover, the absorption peak at 3525 cm^−1^ is enhanced due to the reaction of the carboxyl groups of P_GMA-EDMA_@MIPs with the epoxy groups on the cross-linker molecules, which generates additional free hydroxyl groups.

**Figure 4:**
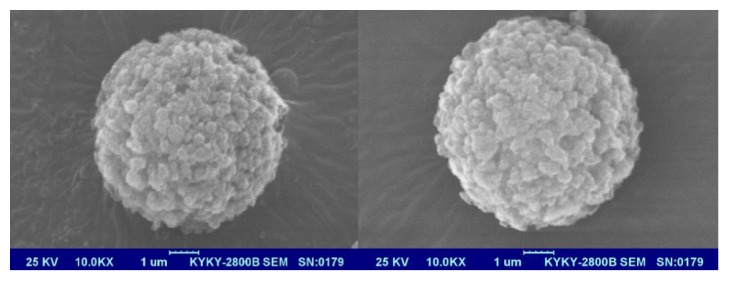
Scanning electron micrographs of P_GMA-EDMA_ microspheres and P_GMA-EDMA_@MIPs.

The SEM analysis of the P_GMA-EDMA_@MIPs and precursor P_GMA-EDMA_ microspheres are depicted in Figure 4. The left image in Figure 4 is an SEM image of the P_GMA-EDMA_ microspheres; the surface of P_GMA-EDMA_ microsphere is divided into uniform pores, formed during the one-step seed swelling and polymerization process used for microsphere preparation. A comparison of the SEM image of the P_GMA-EDMA_ microspheres with that of the P_GMA-EDMA_@MIPs prepared by surface modification (Figure 4, right) reveals the presence of regular gaps in the surface of the P_GMA-EDMA_@MIPs, created upon removal of the template molecule. The particle size of P_GMA-EDMA_@MIPs is about 5 *μ*m and its surface is porous.

The P_GMA-EDMA_ microspheres and P_GMA-EDMA_@MPS and P_GMA-EDMA_@MIPs microspheres were characterized by elemental analysis. The results of these measurements are listed in Table 1. The elemental analysis reveals an increase in carbon content of P_GMA-EDMA_@MPS to the starting material P_GMA-EDMA_, indicating a successful grafting of MPS to the surface of the microspheres. The data for the P_GMA-EDMA_@MIPs indicates an increase in both nitrogen and carbon composition. This increase in N/C content is consistent with the successful grafting of the cross-linking agent EGDE to the surface of the microspheres.

Table 2 shows the specific surface area, pore volume, and average pore size of P_GMA-EDMA_, P_GMA-EDMA_@MIPs, and P_GMA-EDMA_@NIPs from nitrogen adsorption-desorption analysis. As can be seen from (Table 2), the specific surface area of P_GMA-EDMA_@MIPs decreases markedly with respect to P_GMA-EDMA_, which is due to the fact that P_GMA-EDMA_@MIPs is based on P_GMA-EDMA_ to prepare imprinted polymer. P_GMA-EDMA_@MIPs have larger specific surface area, pore volume, and average pore size than P_GMA-EDMA_@NIPs. The results showed that the different adsorption properties of P_GMA-EDMA_@MIPs and P_GMA-EDMA_@NIPs could not only be completely attributed to the difference in morphology but also be related to the imprinting process that produced specific recognition sites. Larger pore volume and average pore size provide complementary spatial structures for selective recognition of template molecules and competitors with P_GMA-EDMA_@MIPs.

### 3.3. Absorption Performance

#### 3.3.1. Absorption Capacity and Kinetics

The adsorption isotherm data of the P_GMA-EDMA_@MIPs were analyzed using Scatchard [28] model and processed according to the following equation:

(6) $$\begin{array}{c} \frac{Q}{C_{e}}=-\frac{Q}{K_{d}}+\frac{Q_{max}}{K_{d}} \end{array}$$

where* Q* and* Q*_*max*_ are the experimental adsorption capacity to the template ENR(mg/g) and the theoretical maximum adsorption capacity of the polymer (mg/g), respectively;* C*_*e*_ is the concentration of ENR in equilibrium in solution (mg/L) after adsorption and* K*_*d*_ is the dissociation constant (mg/L).

**Figure 5:**
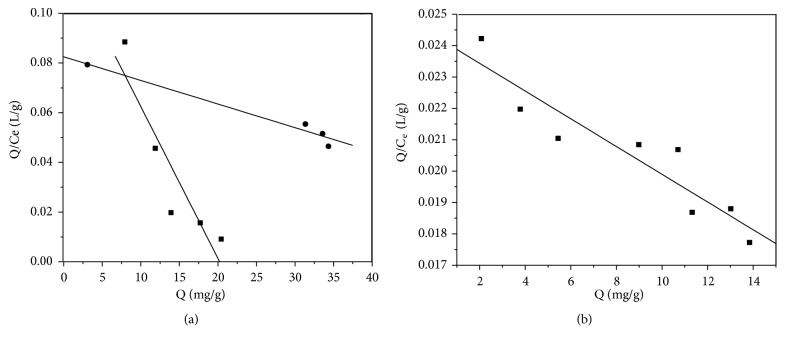
Scatchard analysis plot of the binding of ENR to the P_GMA-EDMA_@MIPs (a) and P_GMA-EDMA_@NIPs (b).

The Scatchard model was implemented on the adsorption isotherm data of the P_GMA-EDMA_@MIPs and P_GMA-EDMA_@NIPs and the Scatchard figure created by plotting* Q* against* Q/Ce* (Figure 5). The Scatchard analysis curve of the P_GMA-EDMA_@MIPs consists of two lines with different slopes, while the Scatchard analysis curve of the P_GMA-EDMA_@NIPs is in a straight line, suggesting the fact that while the P_GMA-EDMA_@NIPs only has a single binding site for ENR, P_GMA-EDMA_@MIPs exhibits two distinct binding sites. One of the binding sites of the P_GMA-EDMA_@MIPs is a nonspecific adsorption site similar to that in P_GMA-EDMA_@NIPs formed by hydrophobic, hydrogen bonds and physical adsorption, while the other is the high affinity specific recognition site created by the imprinted hole. The additional binding site of the P_GMA-EDMA_@MIPs is responsible for the higher adsorption capacity and better selectivity of this system relative to the P_GMA-EDMA_@NIPs.

The values of* K*_*d*_ and* Q*_*max*_ are calculated from the slope and intercept of the linear segments in the Scatchard plots, respectively. The parameters defining P_GMA-EDMA_@MIPs were obtained from the slope and intercept of Scatchard curve, and the results are shown in (Table 3). The* K*_*d*_ and* Q*_*max*_ values were similarly calculated for the P_GMA-EDMA_@NIPs to be 2261.60 mg/L and 55.00 mg/g, respectively.

**Figure 6:**
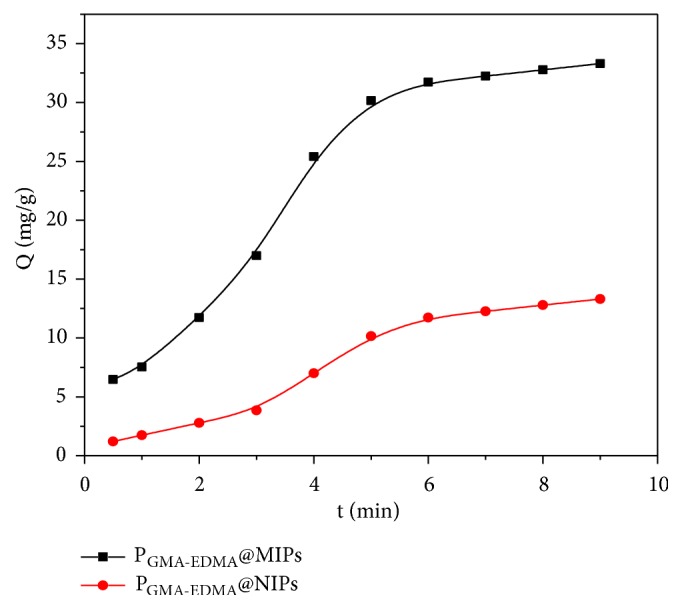
Dynamic adsorption curves of P_GMA-EDMA_@MIPs and P_GMA-EDMA_@NIPs.

Plots of the adsorption kinetics of the P_GMA-EDMA_@MIPs in 2 mM solution of ENR are shown in Figure 6. A rapid adsorption of ENR by the P_GMA-EDMA_@MIPs up to 90.5% of the adsorption equilibrium is noted within the first 5 min, after which a significant decrease in the adsorption rate occurs. The adsorption equilibrium reached at 6 min. Conversely, adsorption by the P_GMA-EDMA_@NIPs was slow within the first 3 min, where the driving force of adsorption has non-covalent interactions between ENR and P_GMA-EDMA_@NIPs. The enhanced binding of ENR by P_GMA-EDMA_@MIPs is dependent on the interaction between ENR and the imprinted holes of the P_GMA-EDMA_@MIPs generated on the surface of microspheres during preparation. From the kinetic data, it is noted that the specific adsorption by P_GMA-EDMA_@MIPs occurs largely in the initial stage of adsorption.

**Figure 7:**
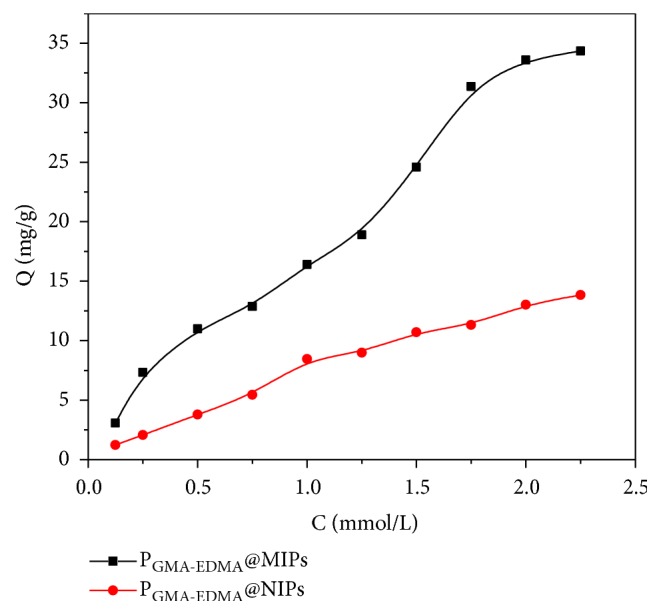
Adsorption isotherm of P_GMA-EDMA_@MIPs and P_GMA-EDMA_@NIPs.

The adsorption isotherm of ENR in the range of 0.125-2.25 mM was obtained by static equilibrium adsorption. The absorption curves showed in Figure 7 show that the adsorption of P_GMA-EDMA_@MIPs gradually increased with increases in ENR concentration, while rapid saturation was observed in the adsorption by P_GMA-EDMA_@NIPs. Adsorption by the P_GMA-EDMA_@MIPs was significantly greater than that of P_GMA-EDMA_@NIPs at a given concentration. The low adsorption capacity of the P_GMA-EDMA_@NIPs was attributed to the weak interactions that form between the P_GMA-EDMA_@NIPs and substrate; substrate binding interactions were derived from the nonspecific adsorption of the polar groups on the P_GMA-EDMA_@NIPs surface to ENR. In addition to nonspecific adsorption interactions, P_GMA-EDMA_@MIPs also contained holes matching the spatial structure and complementing the functional groups of ENR. The holes in the P_GMA-EDMA_@MIPs surface had a memory function for the ENR molecule, and the difference in the adsorption amount of the P_GMA-EDMA_@MIPs and P_GMA-EDMA_@NIPs polymers was largely attributed to the specific adsorption of these holes.

#### 3.3.2. Absorption Selectivity

**Figure 8:**
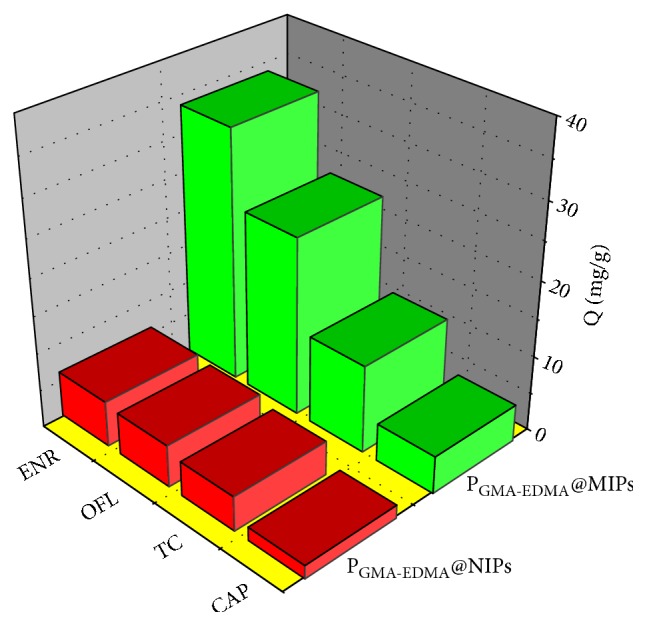
Binding isotherms of ENR, OFL, TC, and CAP on the P_GMA-EDMA_@MIPs and P_GMA-EDMA_@NIPs.

As it can be seen from the data presented in Table 4 and Figure 8, the distribution coefficient* K*_*d*_, selectivity coefficient* k*, imprinting factor *α*, and relative selectivity coefficient *k*′ of adsorbent can be determined via competitive binding experiments. As predicted, the adsorption of ENR and its structural analogues by the P_GMA-EDMA_@MIPs was greater than that by the P_GMA-EDMA_@NIPs. The selectivity coefficient (*k*) defines the selectivity of an absorber over the template molecule. The* k* values of the P_GMA-EDMA_@MIPs were all greater than the corresponding values for the P_GMA-EDMA_@NIPs, suggesting a higher affinity of the P_GMA-EDMA_@MIPs for the template's structural analogues than for other types of antibiotic [29]. Moreover, the relative selectivity coefficient *k*′ values were all greater than 1, indicating that after removal of the template molecule, holes and special imprinting sites were formed on the polymer surface complementing the shape and functional groups of the template molecule. Among ENR and its structural analogues, P_GMA-EDMA_@MIPs has the largest imprinting factor *α* value for ENR, which indicates that P_GMA-EDMA_@MIPs has stronger affinity and excellent selectivity for ENR.

### 3.4. Analysis of Real Samples

**Figure 9:**
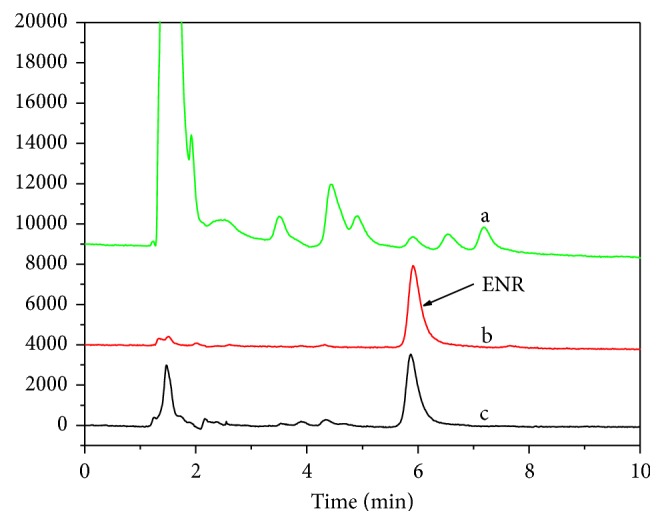
The chromatogram of milk samples: (a) initial milk sample, (b) sample after P_GMA-EDMA_@MIPs treatment, and (c) sample after C_18_-SPE treatment.

Solid phase extraction is afforded via a four-step process: activation, loading, leaching, and eluting. Using water (3 mL) as leachate effectively removes the endogenous components of biological samples, as demonstrated in Figure 9. Following C_18_ extraction, the variance in the recovery of interfering substances was attributed to the type of nonspecific interaction between the different components in the sample matrix and the C_18_ adsorbent, such as the difference between hydrophobic versus hydrophilic interactions. In molecularly imprinted polymer solid-phase extraction (MISPE), the P_GMA-EDMA_@MIPs has better selectivity for the target substrate, resulting in high recovery and a purer extraction.

A standard curve for ENR detection was established over the concentration range 100.0~900.0 *μ*g/L to yield an expression of y=132741x+740.35 and a correlation coefficient of R=0.9986. Milk samples were spiked with ENR standard solutions with concentrations of 100 *μ*g/L, 200 *μ*g/L, and 500 *μ*g/L. As demonstrated in Table 5, the rate of recovery of the ENR from these samples measured between 94.6 and 109.6% upon extraction with the P_GMA-EDMA_@MIPs, with a relative standard deviation (RSD) between 1.1 and 3.2%. The minimum limit of detection (LOD) was determined to be 10 *μ*g/L by tripling the signal-to-noise ratio. (Table 6) summarizes the results of the existing reports on the detection of ENR in the milk samples by different types of ENR imprinted materials. These results demonstrate a high recovery rate for methodology of this study.

## 4. Conclusions

A novel core-shell P_GMA-EDMA_@MIPs was prepared for simultaneous separation and enrichment of four FQs in milk samples. As P_GMA-EDMA_ is easy to modify, stable physicochemical and thermal stability, easily controllable synthesis conditions, low cost, small nonspecific adsorption, and so on, the prepared P_GMA-EDMA_@MIPs had the advantages of stable properties, high selectivity, and recovery rate. P_GMA-EDMA_@MIPs have been successfully applied to the enrichment and separation of FQs in milk samples. This work provides a versatile approach for fabricating well-constructed core-shell P_GMA-EDMA_@MIPs particles for rapid enrichment and highly selective separation of target molecules in real samples.

## Figures and Tables

**Table 1:** Elemental analysis results of P_GMA-EDMA_@MIPs.

Samples	Elemental composition (%)		
	C	N	H
P_GMA-EDMA_	54.95	0.329	6.599
P_GMA-EDMA_@MPS	55.48	0.233	6.442
P_GMA-EDMA_@MIPs	55.87	0.506	5.599

**Table 2:** Comparison of P_GMA-EDMA_ and P_GMA-EDMA_@MIPs from nitrogen adsorption-desorption analysis.

Sample	Surface area /(m^2^*∙*g^−1^)	Pore Volume /(cm^3^*∙*g^−1^)	Average Pore Size /(nm)
P_GMA-EDMA_	150.58	0.930	11.92
P_GMA-EDMA_@MIPs	103.43	0.72	24.92
P_GMA-EDMA_@NIPs	85.02	0.56	8.60

**Table 3:** The results of the Scatchard analysis of P_GMA-EDMA_@MIPs.

Binding site	Linear equation	*K*_d_ (mg/L)	*Q*_max_ (mg/g)
Low affinity	*Q*/*C*_e_ =-0.0009511*Q*+0.0825(R=0.9842)	1051.43	86.74
High affinity	*Q*/*C*_e_=-0.00429*Q*+0.1203 (R=0.9183)	233.10	28.05

**Table 4:** The selective coefficient of the P_GMA-EDMA_@MIPs and P_GMA-EDMA_@NIPs.

Analyte	*Q* (mg/g)		*K*_d_ (mL/g)		*k*		*α*	*k*′
	MIPs	NIPs	MIPs	NIPs	MIPs	NIPs		
ENR	33.58	6.02	46.72	18.12	3.56	3.50	5.58	1.02
OFL	23.70	5.56	32.79	7.69	2. 50	1.49	4.26	1.67
TC	11.67	4.60	13.13	5.17	—	—	2.53	—
CAP	4.97	1.77	7.69	2. 74	—	—	2.80	—

**Table 5:** Recoveries and RSDs of SPE-HPLC method for the spiked milk samples.

	Added concentration/*μ*g L^−1^	Recoveries (%)	RSD (%)
MIP	100	94.6	2.9
	200	95.6	3.2
	500	109.6	1.1
C_18_	100	72.6	4.0
	200	75.6	3.2
	500	78.3	4.4

**Table 6:** Comparison of the ENR-MIPs applied for milk samples' TCs detection with existing reports.

Preparing methods	Test method	Analyte	Linearity range/*μ*g·L^−1^	Limit of detection/*μ*g·L^−1^	Recoveries/%	References
Surface imprinting	HPLC-UV	ENR, PEF	2.5–500	0.7	92.04-98.31	[29]
Surface imprinting	HPLC-UV	ENR, OFL, DAN	50–1000	1.76-12.42	75.6–108.9	[28]
Bulk polymerization	HPLC-UV	ENR, CIP	—	6 5	82.6–93.5	[30]
Sacrificial surface imprinting	HPLC-UV	OFL, ENR, NOR	30–250	—	90.9-102.1	[31]

## Data Availability

The data used to support the findings of this study are included within the paper.
